# GRHL2 induces liver fibrosis and intestinal mucosal barrier dysfunction in non‐alcoholic fatty liver disease via microRNA‐200 and the MAPK pathway

**DOI:** 10.1111/jcmm.15212

**Published:** 2020-04-23

**Authors:** Ying Wang, Zishu Zeng, Lin Guan, Ran Ao

**Affiliations:** ^1^ Department of Gastroenterology the First Affiliated Hospital of China Medical University Shenyang China

**Keywords:** Grainyhead‐like 2, intestinal mucosal barrier dysfunction, liver fibrosis, MAPK signalling pathway, microRNA‐200, non‐alcoholic fatty liver disease, sirtuin‐1, transcription factor

## Abstract

Non‐alcoholic fatty liver disease (NAFLD) serves as the most common subtype of liver diseases and cause of liver dysfunction, which is closely related to obesity and insulin resistance. In our study, we sought to investigate effect of transcription factor grainyhead‐like 2 (GRHL2) on NAFLD and the relevant mechanism. NAFLD mouse model was established with a high‐fat feed. Then, serum was extracted from NAFLD patients and mice, followed by ectopic expression and depletion experiments in NAFLD mice and L02 cells. Next, the correlation between GRHL2 and microRNA (miR)‐200 and between miR‐200 and sirtuin‐1 (SIRT1) was evaluated. The observations demonstrated that miR‐200 and GRHL2 were overexpressed in the serum of NAFLD patients and mice, while SIRT1 was poorly expressed. GRHL2 positively regulated miR‐200 by binding to miR‐200 promoter region, which negatively targeted SIRT1. The inhibition of miR‐200 and GRHL2 or SIRT1 overexpression lowered HA and LN in mouse liver tissue, occludin and ZO‐1 in mouse small intestine tissue, TNF‐α and IL‐6 in mouse serum, glucose, total cholesterol (TC), triglyceride (TG), aspartate aminotransferase (AST) and alanine aminotransferase (ALT) in mouse serum, and also inhibited liver fibrosis and intestinal mucosal barrier dysfunction. Meanwhile, GRHL2 induced activation of MAPK signalling pathway in NAFLD mice. Collectively, GRHL2 played a contributory role in NAFLD by exacerbating liver fibrosis and intestinal mucosal barrier dysfunction with the involvement of miR‐200‐dependent SIRT1 and the MAPK signalling pathway.

## 1 INTRODUCTION

As a new health problem, non‐alcoholic fatty liver disease (NAFLD) influences one‐third of adults and an increasing number of children in developed countries.^1^ NAFLD involves a spectrum of liver injuries including steatosis and steatohepatitis with or without fibrosis.^2^ NAFLD affected nearly one‐third of people, and patients with NAFLD‐related terminal or deteriorative liver diseases have become one of the major groups receiving liver transplantation.^3^ Although lots of advances have been made in the prognosis for NAFLD, the pathogenesis of NAFLD remains poorly identified. Fibrosis is one of the causes for NAFLD,^4^ and the degree of liver fibrosis is connected with therapeutic decisions or disease outcomes.^5^ Intestinal mucosal barrier dysfunction is also intricately relevant to liver diseases, and progression of NAFLD might be attributed to impaired gut‐liver axis.^6^ Thus, the current study was designed to investigate new molecular mechanisms and explore new targets for NAFLD and associated disorders.

The transcription factor including sterol regulatory element‐binding protein‐1c was previously found to modulate lipogenesis and insulin sensitivity and was experimentally involved in NAFLD.^7^ One of the transcription factors, grainyhead‐like 2 (GRHL2), was found to be involved in hepatocytic differentiation potential of liver stem/progenitor cells.^8^ GRHL2 was also reported to positively target miR‐200B/200A directly at their promoters.^9^ miR‐200 was identified as a prognostic biomarker in different tumour types.^10^ Furthermore, sirtuin 1 (SIRT1) has been implied to control cellular processes, including metabolism and stress responses by deacetylating a lot of cellular proteins, such as histones, transcription factors, DNA repair proteins and autophagy factors.^11^ Plasma level of SIRT1 was also identified to be associated with NAFLD in obese patients.^12^ Interestingly, the regulatory role of miR‐200a on SIRT1 in mammary epithelial cells was previously investigated.^13^ GRHL2 was found to stimulate MAPK signalling pathway in oral cancer development.^14^ Moreover, Ras‐mitogen‐activated protein kinase (MAPK) signalling pathway was found to exert influence on NAFLD.^15^

The before‐mentioned findings trigger a possible mechanism that GRHL2 might be involved in NAFLD via miR‐200‐targeted SIRT1 and MAPK signalling pathway. Thus, this study aimed to verify whether this regulatory network was involved in the liver fibrosis and intestinal mucosal barrier dysfunction of NAFLD.

## 2 MATERIALS AND METHODS

### 2.1 Ethics statement

All participants signed the informed written consents. The current study was approved by the Ethics Committee of the First Affiliated Hospital of China Medical University (Approval Number: 201511016) and carried out in accordance with the *Declaration of Helsinki*. All animal experiments in this study strictly adhered to the Guide for the Care and Use in of Laboratory Animal by National Institutes of Health under a protocol approved by the Laboratory Animal Care and Use Committee of the First Affiliated Hospital of China Medical University (Approval Number: 201903011).

### 2.2 Study subjects

The study enrolled 57 NAFLD patients (aged from 22 to 59 years with a mean age of 41.30 ± 7.93 years) who received specialist outpatient service in the First Affiliated Hospital of China Medical University from January 2016 to December 2017. Among them, 35 were males and 22 were females. All patients met the B‐ultrasonography diagnostic criteria for NAFLD. And the patients with hypertension, coronary heart disease, diabetes, viral hepatitis, history of alcoholism or any hereditary diseases were excluded. According to B‐mode ultrasound images, the number of mild, moderate and severe cases was 17, 22 and 18, respectively.

Moreover, thirty healthy people, without alcoholism, who had a check‐up in the First Affiliated Hospital of China Medical University from January 2016 to December 2017 were enrolled and served as control. These people, comprising 18 males and 12 females, aged from 29 to 61 years with a mean age of 42.93 ± 8.44 years. All the healthy people had normal liver and kidney function as well as normal electrocardiogram. Likewise, people with a medical history of hypertension, hyperlipidaemia, diabetes, hepatitis and neurological diseases were excluded.

Blood sample (5 mL) was collected from limosis patients in the early morning and healthy people during the check‐up, respectively. Then, the blood samples were added into the tube containing sodium citrate solution, followed by a 15‐min centrifugation at 3000 rpm and 4°C to isolate plasma from cells. Lastly, the plasma was stored at −80°C for subsequent experiments.

### 2.3 Animal models and treatment

C57BL/6 mice were purchased from the Animal Center of Academy of Military Medical Sciences. Eight mice were fed with a standard diet (326 kCal for 100 g). Mouse model of NAFLD (n = 72) was successfully established using 4‐week high‐fat feeding (20% lard, 4% sugar, 15% milk powder, 1% cholesterol and 73% normal diet; 453 kCal for 100 g), followed by transduction with the plasmids. Animal diets were purchased from Changchun Yisi Experimental Animal Technology Co., Ltd. Then, the mice were killed after anaesthesia using 10% sodium pentobarbital. Serum from the inferior vena cava, liver tissues and small intestine tissues were collected. An automatic biochemical analyzer (Beckman, Fullerton, CA, USA) was adopted to measure the glucose, total cholesterol (TC), triglyceride (TG), aspartate aminotransferase (AST) and alanine aminotransferase (ALT) in the plasma, followed by model identification using HE staining. Pure water without endotoxin was applied to dilute nucleic acid (12.5 μg) into 1 μg/μL, followed by addition of 12.5 μL water and 10% glucose solution (w/v; 25 μL) with the final volume of 50 μL. EntransterTM‐invivo animal in vivo transduction reagent (25 μL; Engreen) was diluted by 10% glucose solution (25 μL) with the final volume of 50 μL, followed by rapid addition into diluted nucleic acid solution and 15‐min standing at room temperature. The mixture was injected into mice (100 μg nucleic acid and 50 μL transduction reagent every time for each mouse). With 8 untreated modelled mice as model group, NAFLD mice were transduced with miR‐200 inhibitor, NC inhibitor, oe‐GRHL2, oe‐NC for oe‐GRHL2, sh‐GRHL2, sh‐NC or oe‐NC for oe‐SIRT1 and oe‐SIRT1 plasmids, with 8 mice for each group.

### 2.4 Cell treatment

Human normal hepatocytes L02 (3142C0001000000077; Cell Bank of Typical Culture Preservation Center) were cultured in RPMI1640 medium containing 10% foetal bovine serum (FBS) (12633012, Shanghai Haoran Biological Technology Co., LTD.) at 37°C with 5% CO_2_. In line with the instruction of Lipofectamine 2000 kit (Invitrogen, Carlsbad, CA, USA), cells at the logarithmic phase growth were transduction with plasmids containing overexpression (oe)‐GRHL2, oe‐negative control (NC), short hairpin (sh)‐GRHL2, sh‐NC, miR‐200 mimic and NC mimic alone or in combination. After another 4 hours of incubation, G418 (400 μg/mL) was added. The culture medium was changed every three days, and G418 was reduced to maintain screening until stably transduced cells were harvested.

### 2.5 Chromatin immunoprecipitation (ChIP) assay

Cells were fixed using formaldehyde for 10 minutes, and then, chromosome DNA fragmentation was performed using ultrasonic treatment (10 seconds on, 10 seconds off) for 15 times, followed by 12 000 × *g* centrifugation at 4°C for 10 minutes. The supernatants were collected and assigned to two tubes, with addition of the negative control immunoglobulin G antibody (IgG; ab172730, 1:1000, Abcam) and the target protein‐specific anti‐GRHL2 (ab86611, 1:1000, Abcam) antibody, respectively, followed by overnight incubation at 4°C. Protein agarose/Sepharose was used to precipitate DNA‐protein complex. After a centrifugation at 12 000 × *g* for 5 minutes, the supernatant was discarded. Non‐specific DNA‐protein complex was washed to remove. After de‐crosslinked at 65°C overnight, the DNA fragments were then collected and purified by phenol chloroform extracting method, and the detection of combination between miR‐200 and GRHL2 was performed using reverse transcription quantitative polymerase chain reaction (RT‐qPCR) based on the specific primers in miR‐200 promoter region.

### 2.6 Dual‐luciferase reporter gene assay

The 3’‐untranslated region (3'UTR) of SIRT1 was artificially synthesized and inserted into the pMIR‐reporter (Beijing Huayueyang Biotechnology Co., Ltd., Beijing, China) using restriction endonuclease SpeI and Hind III. The complementary sequence mutation site of seed sequence was designed on the wild type (WT) of SIRT1 and miR‐200, respectively, and then, target fragments were constructed into the pMIR‐reporter using T4 DNA ligase. The correctly sequenced WT and MUT plasmids were cotransfected with mimic NC or miR‐200 mimic into HEK‐293T (Cell Resource Center, Shanghai Institute of Life Sciences, Shanghai Academy of Chinese Sciences). After transfected for 48 hours, the cells were collected and lysed, and the luciferase activity was measured using the luciferase assay kit (K801‐200, BioVision Technologies) and Glomax20/20 luminometer (Promega).

Promoter region of WT‐ and MUT‐miR‐200 was ligated into pGL3‐Basic vector (Promega) to construct recombinant vector of WT‐miR‐200 promoter and MUT‐miR‐200 promoter. HEK‐293 cells were seeded into 24‐well plates at the density of 3 × 10^4^ cells/well. The WT‐miR‐200 promoter and MUT‐miR‐200 promoter were cotransfected with oe‐NC and oe‐GRHL2 into HEK‐293T cells, respectively. After 48 hours of transfection, cells were collected and lysed, and the luciferase activity was measured using the luciferase assay kit (K801‐200, BioVision Technologies) and Glomax20/20 luminometer (Promega).

### 2.7 Haematoxylin‐eosin staining

Liver tissues were extracted from mice and fixed, followed by paraffin embedding and section (4 µm). Then, the sections were dewaxed with xylene and gradient ethanol: xylene (I) for 5 minutes, toluene (II) for 5 minutes, 100% ethanol for 2 minutes, 95% ethanol for 1 minutes, 80% ethanol for 1 minute and 75% ethanol for 1 minute. The tissues were then stained with haematoxylin for 5 minutes, followed by differentiation of ethanol hydrochloride for 30 seconds. The sections were soaked in tap water for 15 minutes or warm water (about 5°C; 5 minutes), followed by eosin staining for 2 minutes. Then, routine dehydration, clearing and mounting were performed: 95% ethanol (I) for 1 minute, 95% ethanol (II) for 1 minute, 100% ethanol (I) for 1 minute, 100% ethanol (II) for 1 minute, toluene carbonate (3:1) for 1 minute, toluene (I) for 1 minute and xylene (II) for 1 minute. Next, the sections were mounted by neutral resin, which were finally observed and photographed using microscope (XSP‐8CA, Shanghai Optical Instrument Factory) for histological changes of liver tissues.

### 2.8 Masson's trichrome staining

Sections were dewaxed and rehydrated conventionally, stained with Weigert's haematoxylin for 5‐10 minutes and rinsed under water. Ethanol hydrochloride differentiation solution was used to differentiate the tissues, followed by Ponceau 3R staining for 5‐10 minutes. Later, the sections were immersed in 1% phosphomolybdic acid solution for about 5 minutes until the collagen fibre or background was colourless and the myelin sheath was red. Subsequently, sections were counter‐stained in aniline blue for 5 minutes and immersed in 1% acetic acid for 1 minute, followed by dehydration using 95% ethanol multiple times. After dehydration with absolute ethanol, and permeabilization with xylene for 10 minutes, the sections were sealed with neutral resin. Finally, these sections were observed under an inverted microscope (XSP‐8CA, Shanghai Optical Instrument Factory).

### 2.9 Oil Red O staining

Oil Red O staining was performed using the procedure described previously.^16^ Briefly, sections were incubated in distilled water for 1 minute and in 100% propylene glycol (Polyscientific) for 2 minutes. Some sections were transferred to Oil Red O for 36‐hours incubation at room temperature. The other sections were incubated for 1 minute in 85% propylene glycol, then stained with haematoxylin and mounted with gelatin mounting medium.

### 2.10 RT‐qPCR

Total RNA was extracted using TRIzol (Invitrogen, Carlsbad, CA) and reverse‐transcribed into cDNA by reverse transcription kit (RR047A, Takara). RT‐qPCR was performed using TaqMan MicroRNA Assay and TaqMan^®^ Universal PCR Master Mix. SYBR Premix EX Taq kit (RR420A, Takara) and ABI7500 Real‐Time PCR System (7500, ABI) were used to perform RT‐qPCR. Three replicates were set for all samples. Primer sequences were synthesized in Shanghai Sangon Biotechnology (Table 1). Glyceraldehyde‐3‐phosphate dehydrogenase (GAPDH) and U6 served as internal references for mRNA for miRNA, respectively. 2^−ΔΔCt^ method was adopted to calculate the relative expression of target genes as previously described.^17^

**TABLE 1:** Primer sequences for RT‐qPCR

Target gene	Primer sequence	
miR‐200 (human)	F	ACACTCCAGCTGGGTAACACTGTCTGGTAACG
	R	CTCAACTGGTGTGGTGGAGTCGGCAATTGAGTTGAGACATCGTT
miR‐200 (mice)	F	AAGCGCCTTAACACTGTCTGG
	R	CAGTGCAGGGTCCGAGGT
GRHL2 (mice)	F	5’‐TTTGGTCCAACACCGTCTA‐3’
	R	5’‐CACTGGCAGCCCATACTT‐3’
SIRT1 (mice)	F	5’‐CGGCTACCGAGGTCCATATAC‐3’
	R	5’‐ACAATCTGCCACAGCGTCAT‐3’
U6 (human)	F	5’‐ CGCTTCGGCAGCACATATACTA‐3’
	R	5’‐ CGCTTCACGAATTTGCGTGTCA‐3’
U6 (mice)	F	5’‐ TCGCACAGACTTGTGGGAGAA‐3’
	R	5’‐ CGCACATTAAGCCTCTATAGTTACTAGG‐3’
GAPDH (mice)	F	5’‐ TTAGCACCCCTGGCCAAGG‐3’
	R	5’‐ CTTACTCCTTGGAGGCCATG‐3’

Abbreviations: F, forward; GAPDH, glyceraldehyde‐3‐phosphate dehydrogenase; GRHL2, grainyhead‐like transcription factor 2; miR‐200, microRNA 200 S100B; R, reverse, RT‐qPCR, reverse transcription quantitative polymerase chain reaction; SIRT1, sirtuin 1.

### 2.11 Western blot analysis

The tissues were shredded and then lysed. Total proteins were extracted using radioimmunoprecipitation assay (RIPA) lysis buffer containing phenylmethylsulphonyl fluoride (PMSF), followed by centrifugation at 8000 *g* for 10 minutes after incubated on the ice for 30 minutes. BCA protein assay kit was employed to detect the protein concentration. Then, proteins were separated by sodium dodecyl sulphate‐polyacrylamide gel electrophoresis and then transferred onto polyvinylidene fluoride membrane by wet transfer method. The membrane was blocked with 5% skim milk powder for 1 hour and incubated overnight with the following primary rabbit antibodies which were diluted with milk: GRHL2 (1:1000, ab136682), SIRT1 (1:1000, ab12193), laminin (LN; 1:1000, ab191904), hyaluronic acid (HA; 1:4000, ab9110), occludin (1:1000, ab216327), ZO‐1 (1:3000, ab96587), extracellular signal–regulated kinase (ERK; 1:1000, ab53277), phosphorylated (p)‐ERK (1:1000, ab79483), p38 (1:1000, ab225534), p‐p38 (1:1000, ab47363), c‐Jun NH2‐terminal kinase (JNK; 1:1000, ab179461), p‐JNK (1:10 000, ab124956) and GAPDH (1:10 000, ab181602). The above‐mentioned antibodies were purchased from Abcam (Cambridge, UK). After rinsed with TBST for 3 times (10 min/time), the horseradish peroxidase–labelled secondary goat anti‐rabbit IgG (1:2000, ab6721, Abcam) was then added to the membrane and incubated for 1 hour. Next, the membrane was washed with TBST and electrochemiluminescence was applied (ECL; BB‐3501) to visualize image. Bio‐Rad gel image analysis system was employed to photograph, and Quantity one v4.6.2 software was used to analyse relative protein expression which was expressed as the ratio of the grey value of the target band to that of GAPDH.

### 2.12 ELISA

ELISA kit (RAB0287; Sigma) was coated with specific IL‐6 antibody. After the addition of plasma, specific biotinylated antibody IL‐6 was supplemented to combine IL‐6 in plasma, followed by incubation at room temperature. The unconjugated biotinylated antibody was eliminated, and streptavidin‐peroxidase conjugate was then added. After termination of the reaction, the uncoupled conjugate was washed away. Tetramethylbenzidine (TMB) contained in TMB substrate could be induced to blue conjugate by streptomyces and lignin peroxidase, and turned yellow after addition of acidic stop solution. The density of yellow and the content of IL‐6 in the sample are in proportion to the bottom of the kit. Then, the optical density (OD) value was detected using a microplate reader. TNF‐α was quantified as above described with the ELISA kit (RAB0477, Sigma).

### 2.13 Statistical analysis

All data were processed and analysed using SPSS 21.0 statistical software (IBM Corp.). Measurement data were expressed as mean ± standard deviation. For data conformed to normal distribution and homogeneity of variance, unpaired *t* test was adopted to analyse the differences between, while one‐way analysis of variance (ANOVA) was utilized to compare data among multiple groups, followed by Tukey's post hoc test. *P* < .05 represented statically significant.

## 3 RESULTS

### 3.1 MiR‐200 was overexpressed in serums of NAFLD patients and mice

**FIGURE 1:**
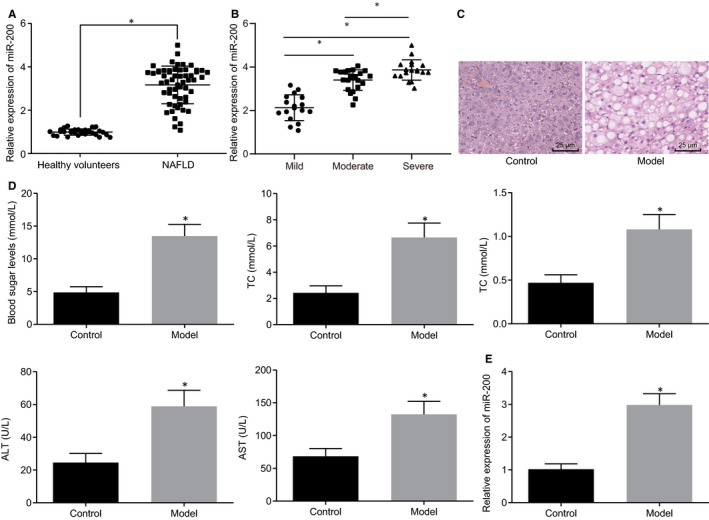
miR‐200 overexpression was observed in serum of NAFLD patients and mice. A, miR‐200 expressions in serums of 57 NAFLD patients and 30 healthy volunteers determined by RT‐qPCR (normalized to U6). B, Correlation of miR‐200 expression in serum with severity of NAFLD patients as determined by RT‐qPCR. C, HE staining results of histological change in mouse liver tissues (×400). D, Glucose, TC, TG, AST and ALT contents in NAFLD mice and control mice. E, MiR‐200 expression in the serum of mice measured using RT‐qPCR (normalized to U6) (n = 8). *P < .05 *vs.* healthy volunteers or control mice. Measurement data were expressed as mean ± standard deviation and analysed by unpaired *t* test

RT‐qPCR was adopted to measure miR‐200 expression in serums of 57 NAFLD patients and 30 healthy volunteers, which showed that miR‐200 expression was elevated in the serums of NAFLD patient (Figure 1A, *P* < .05). The correlation between serum miR‐200 expression and the severity of NAFLD in patients was analysed by RT‐qPCR, which displayed that serum miR‐200 expression was positively correlated with the severity of NAFLD (Figure 1B, *P* < .05). As shown in Figure 1C, D, HE staining results revealed that compared with control mice, NAFLD mice were characterized by hepatocellular swelling with cytoplasmic vacuoles in hepatocytes. And part of the hepatocytes was damaged with inflammatory cell infiltration. In addition, serum glucose, TC, TG, AST and ALT contents were elevated in NAFLD mice (all *P* < .05), which indicated that the NAFLD mouse models were successfully established. Subsequently, the serum expression of miR‐200 in NAFLD mouse models and control mice was detected using RT‐qPCR (Figure 1E), and the results that the serum expression of miR‐200 potently rose in NAFLD mice were revealed. Thus, either the serum of NAFLD patients or that of NAFLD mice exhibited highly expressed miR‐200.

### 3.2 Silencing of miR‐200 ameliorated liver fibrosis and intestinal mucosal barrier dysfunction in NAFLD mice

**FIGURE 2:**
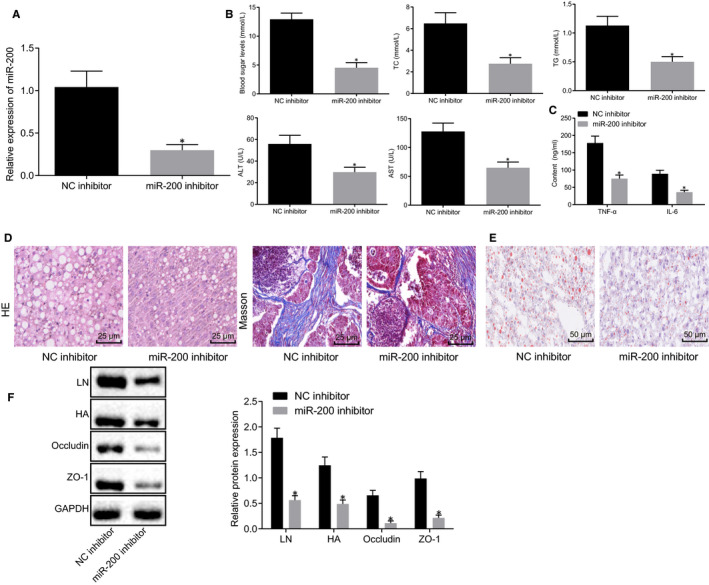
MiR‐200 down‐regulation alleviated liver fibrosis and intestinal mucosal barrier dysfunction in NAFLD mice. NAFLD mice were transduced with miR‐200 inhibitor or NC inhibitor plasmids. A, MiR‐200 expression in liver tissues of NAFLD mice determined using RT‐qPCR (normalized to U6). B, Blood glucose, TC, TG, ALT and AST in the serum of NAFLD mice. C, Expression of inflammatory factors (TNF‐α and IL‐6) in the serum of NAFLD mice determined using ELISA. D, Liver fibrosis of liver tissue detected by HE staining and Masson staining (×400). E, Lipid accumulation in liver tissue determined using Oil Red O staining (×200). F, Western blot analysis of protein expression of LN and HA in liver tissue, occludin and ZO‐1 in small intestine tissues of NAFLD mice. *P < .05 *vs.* NAFLD mice injected with NC inhibitor. Measurement data were expressed as mean ± standard deviation and analysed by unpaired *t* test. n = 8

To better elucidate the mechanism underlying the effect of miR‐200 on liver fibrosis and intestinal mucosal barrier dysfunction, liver function indicators, inflammatory factors, liver tissues changes, liver fibrosis and intestinal mucosal barrier dysfunction were detected in NAFLD mice treated with miR‐200 inhibitor. As depicted in Figure 2A, reduction of miR‐200 expression in liver tissues of NAFLD mice was observed in response to miR‐200 inhibitor. Moreover, after treatment of miR‐200 inhibitor, significantly reduced blood glucose, TC, TG, ALT, AST, TNF‐α and IL‐6 were found in serum of NAFLD mice (Figure 2B, C, *P* < .05). Next, the fibrosis and lipid accumulation of liver was determined using HE staining, Masson staining and Oil Red O staining. It was obvious that there were decreases of the fibrous tissue, fibrous hyperplasia and the lipid droplets in the portal area of NAFLD mice with miR‐200 inhibitor (Figure 2D, E). Western blot analysis was conducted to determine the protein expression of liver fibrosis (HA and LN)– and intestinal mucosal barrier function–related proteins (occludin and ZO‐1) in liver tissues and small intestine tissues, respectively. According to the results, LN, HA, occludin and ZO‐1 protein expression dramatically reduced after injection of miR‐200 inhibitor in NAFLD mice (Figure 2F, *P* < .05). Coherently, miR‐200 inhibition might ameliorate liver fibrosis and intestinal mucosal barrier dysfunction in NAFLD mice.

### 3.3 GRHL2 down‐regulated SIRT1 expression by activating miR‐200

**FIGURE 3:**
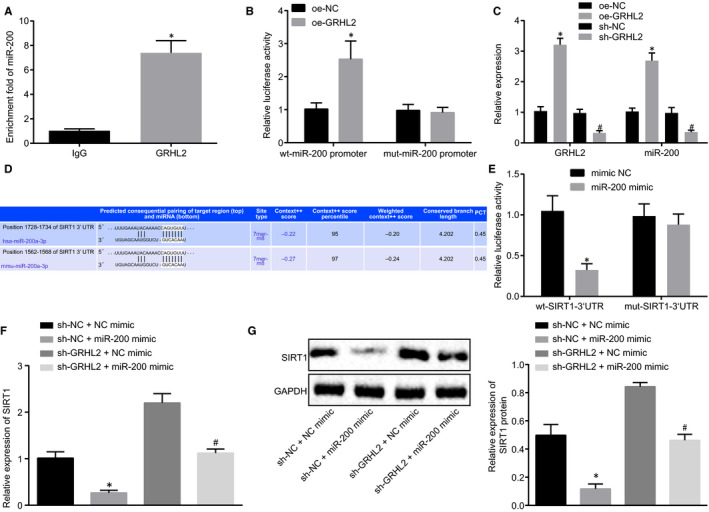
GRHL2 down‐regulated SIRT1 expression by activating miR‐200. A, Enrichment of miR‐200 in miR‐200 promoter region determined using ChIP normalized to IgG. B, The binding relationship between GRHL2 and miR‐200 verified by dual‐luciferase reporter assay. C, MiR‐200 and GRHL2 expression in response to oe‐GRHL2 and sh‐GRHL2 measured using RT‐qPCR. D, Bioinformatics prediction of the binding sites between miR‐200 and SIRT1. E, The luciferase activity of WT‐SIRT1‐3'UTR and MUT‐SIRT1‐3'UTR detected by dual‐luciferase reporter assay. F, SIRT1 mRNA expression in normal human hepatocyte L02 measured using RT‐qPCR normalized to GAPDH. G, SIRT1 protein expression in normal human hepatocyte L02 measured using Western blot analysis normalized to GAPDH. *P < .05 *vs.* IgG, oe‐NC, sh‐NC+NC mimic or mimic NC. ^#^ *P* < .05 *vs.* sh‐NC or sh‐GRHL2+NC mimic. Measurement data were expressed as mean ± standard deviation. Unpaired *t* test was adopted to analyse the differences between two groups with unpaired design, while one‐way ANOVA was utilized to compare data among multiple groups, followed by Tukey's post hoc test. Cell experiments were conducted in triplicates

ChIP assay was firstly conducted to detect the association of GRHL2 to miR‐200 promoter region. Results demonstrated that increased miR‐200 was co‐precipitated by GRHL2, suggesting GRHL2 up‐regulated the enrichment of miR‐200 in miR‐200 promoter region (Figure 3A). Next, dual‐luciferase reporter assay identified intensified luciferase activity in response to cotransfection of oe‐GRHL2 and WT‐miR‐200 promoter (*P* < .05), while no significant disparity was seen after cotransfection of oe‐GRHL2 and MUT‐miR‐200 promoter (*P* > .05; Figure 3B). Further, RT‐qPCR results revealed that GRHL2 and miR‐200 expression in normal human hepatocyte L02 significantly increased in response to oe‐GRHL2, while an opposite trend was seen in response to sh‐GRHL2 (Figure 3C). Taken together, GRHL2 could promote miR‐200 expression by recruiting miR‐200 to miR‐200 promoter region.

Bioinformatics predicted that miR‐200 might target SIRT1 (Figure 3D), which was then verified using dual‐luciferase reporter assay. As documented in Figure 3E, A significant drop of luciferase activity in response to cotransfection of miR‐200 mimic and WT‐SIRT1‐3'UTR was seen (*P* < .05), while no changes were observed after treatment of miR‐200 mimic and MUT‐SIRT1‐3'UTR (*P* > .05). RT‐qPCR and Western blot analysis were then performed to determine SIRT1 expression in normal human hepatocyte L02, and the results revealed obvious decrease in SIRT1 expression in response to sh‐NC+miR‐200 mimic or sh‐GRHL2+miR‐200 mimic compared with sh‐NC+NC mimic or sh‐GRHL2+NC mimic, respectively (Figure 3F, G). The above‐mentioned evidence suggested that GRHL2 down‐regulated SIRT1 expression by activating miR‐200.

### 3.4 SIRT1 overexpression inhibited liver fibrosis and intestinal mucosal barrier dysfunction in NAFLD mice

**FIGURE 4:**
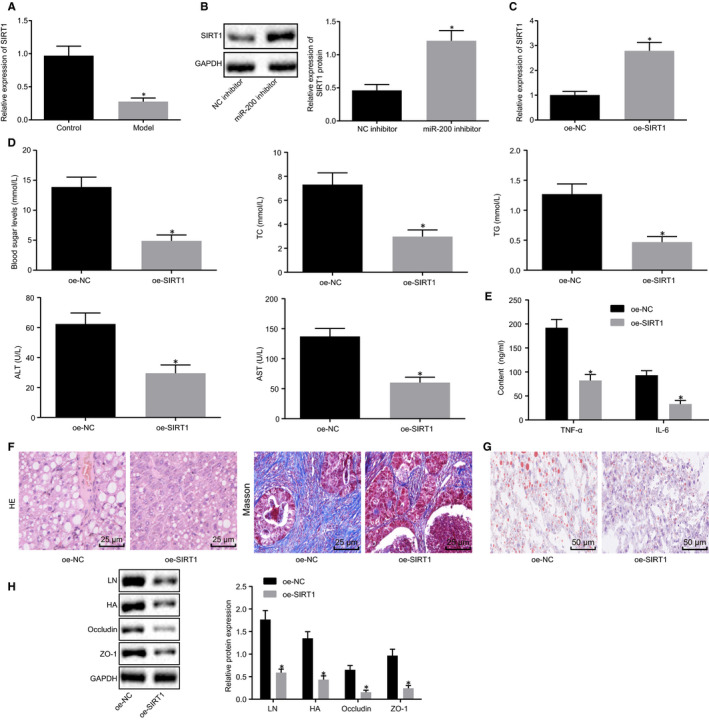
SIRT1 up‐regulation repressed liver fibrosis and intestinal mucosal barrier dysfunction in NAFLD mice. A, SIRT1 mRNA expression in liver tissues of NAFLD mice and normal mice determined by RT‐qPCR normalized to GAPDH. B, SIRT1 protein expression in liver tissues of NAFLD mice transduced with miR‐200 inhibitor plasmids detected by Western blot analysis normalized to GAPDH. NAFLD mice were injected with oe‐NC or oe‐SIRT1. C, SIRT1 expression in liver tissues of NAFLD mice determined by RT‐qPCR normalized to GAPDH. D, Blood glucose, TC, TG, ALT and AST in the serum of NAFLD mice. E, Levels of TNF‐α and IL‐6 in the serum of NAFLD mice measured by ELISA. F, Liver fibrosis in liver tissue determined using HE staining and Masson staining (×400). G, Lipid accumulation in liver tissue measured by Oil Red O staining (× 200). H, Western blot analysis of protein expression of LN and HA in liver tissue, occludin and ZO‐1 in small intestine tissues of NAFLD mice normalized to GAPDH. *P < .05 *vs.* control mice, NAFLD mice injected with NC inhibitor or oe‐NC. Measurement data were expressed as mean ± standard deviation and compared by unpaired *t* test. n = 8

To better elucidate the role of SIRT1 in NAFLD, RT‐qPCR was conducted to measure SIRT1 mRNA expression in liver tissues of mice, which identified the expression of SIRT1 was down‐regulated in liver tissues of NAFLD mice (Figure 4A). Western blot analysis results revealed an obvious rise of SIRT1 protein expression after inhibition of miR‐200, suggesting miR‐200 down‐regulation could increase SIRT1 expression in liver tissues of NAFLD mice.

Subsequently, several parameters about liver fibrosis and intestinal mucosal barrier dysfunction were detected in SIRT1‐overexpressed NAFLD mice. After treatment of oe‐SIRT1, SIRT1 expression increased in liver tissues of NAFLD mice via the results of RT‐qPCR (Figure 4C). As demonstrated in Figure 4D, E, blood glucose, TC, TG, ALT, AST, TNF‐α and IL‐6 were reduced in serum of SIRT1‐overexpressed NAFLD mice. The fibrosis and lipid accumulation of liver was determined using HE staining, Masson staining and Oil Red O staining (Figure 4F, G *, P* < .05). The results exhibited that treatment with oe‐SIRT1 decreased the fibrous tissue, fibrous hyperplasia and the lipid droplets in NAFLD mice. Further, Western blot analysis results revealed that protein expression of LN and HA in liver tissue, occludin and ZO‐1 in small intestine tissues obviously reduced in NAFLD mice after injection of oe‐SIRT1 (Figure 4H). Taken together, SIRT1 overexpression may inhibit liver fibrosis and intestinal mucosal barrier dysfunction in NAFLD mice.

### 3.5 GRHL2 exacerbated liver fibrosis and intestinal mucosal barrier dysfunction in NAFLD mice via activating MAPK signalling pathway

**FIGURE 5:**
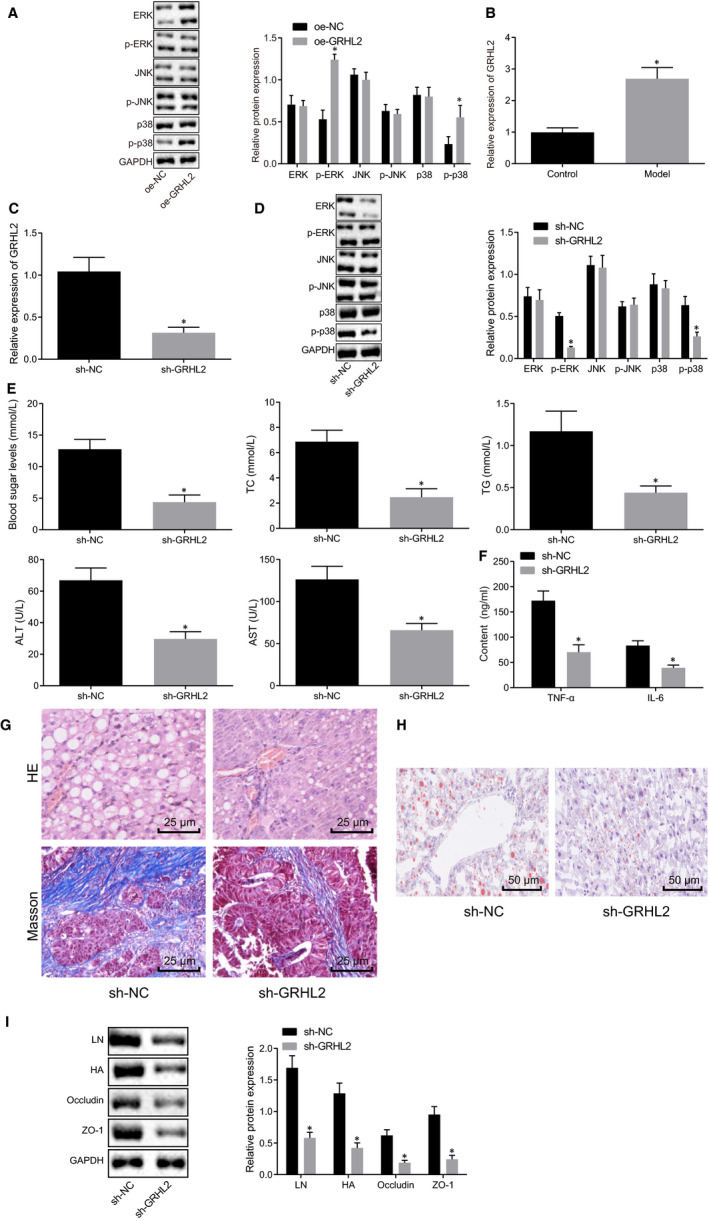
GRHL2 activated MAPK signalling pathway and then worsen liver fibrosis and intestinal mucosal barrier dysfunction in NAFLD mice. A, Expression of MAPK‐related markers in L02 cells determined using Western blot analysis normalized to GAPDH. B, GRHL2 expression observed in liver tissues of NAFLD mice and control mice detected using RT‐qPCR normalized to GAPDH. NAFLD mice were transduced with sh‐NC or sh‐GRHL2 plasmids. C, GRHL2 expression in liver tissues of NAFLD mice tissues using RT‐qPCR normalized to GAPDH. D, Western blot analysis of MAPK‐related markers expressions in liver tissues of NAFLD mice normalized to GAPDH. E, Blood glucose, TC, TG, ALT and AST in serum of NAFLD mice. F, Expression of TNF‐α and IL‐6 in serum of NAFLD mice determined using ELISA. G, Liver fibrosis in liver tissues determined using HE staining and Masson staining (×400). H, Lipid accumulation in liver tissues determined using Oil Red O staining (×200). I, The protein expression of LN and HA in liver tissue, occludin and ZO‐1 in small intestine tissues of NAFLD mice determined by Western blot analysis normalized to GAPDH. *P < .05 *vs.* control mice, or NAFLD mice injected with oe‐NC or sh‐NC. Measurement data were expressed as mean ± standard deviation and compared by unpaired *t* test. n = 8. Cell experiments were performed 3 times independently

GRHL2 was found to activate MAPK signalling pathway in oral cancer, while MAPK signalling pathway was found to be involved in NAFLD.^14^, ^15^ We proposed a hypothesis that GRHL2 might exert influence on NAFLD by activating MAPK signalling pathway. To test this, normal human hepatocyte L02 was transfected with oe‐GRHL2 plasmids and the expression of MAPK‐related markers was measured using Western blot analysis. Compared with cells transfected with oe‐NC plasmids, phosphorylated ERK and phosphorylated p38 expression was elevated in normal human hepatocyte L02 cells transfected with oe‐GRHL2 (*P* < .05), while there were no significant differences in ERK, p38, JNK and phosphorylated JNK expressions (*P* > .05; Figure 5A), suggesting GRHL2 could activate MAPK signalling pathway. Meanwhile, GRHL2 expression in liver tissues of mice was assessed using RT‐qPCR, of which the results indicated a GRHL2 up‐regulation in NAFLD mice (Figure 5B).

To better explore its underlying mechanism, we silenced GRHL2 in vivo to detect to detect liver function indicators, inflammatory factors, liver tissue changes, liver fibrosis and intestinal mucosal barrier dysfunction in non‐alcoholic fatty liver. As demonstrated in Figure 5C, GRHL2 expression was declined in liver tissues of NAFLD mice after silencing GRHL2. In addition, Western blot analysis was adopted to determine the expressions of MAPK‐related markers in liver tissues of NAFLD mice, and the results demonstrated that phosphorylated ERK and phosphorylated p38 expression potently reduced in liver tissues of mice injected with sh‐GRHL2, while no obvious change was seen in ERK, p38, JNK and phosphorylated JNK expression (Figure 5D). Further, significantly reduced blood glucose, TC, TG, ALT, AST, TNF‐α and IL‐6 were found in serum of GRHL2‐slienced NAFLD mice (Figure 5E, F). Next, the fibrosis and lipid accumulation of liver was determined using HE staining, Masson staining and Oil Red O staining (Figure 5G, H). In response to sh‐GRHL2, the fibrous tissue, fibrous hyperplasia and the lipid droplets were decreased. Through Western blot analysis, the protein expressions of LN and HA in liver tissue, occludin and ZO‐1 in small intestine tissues reduced in NAFLD mice after silencing GRHL2 (Figure 5I). Taken together, GRHL2 promoted liver fibrosis and intestinal mucosal barrier dysfunction in NAFLD mice through activation of MAPK signalling pathway.

## 4 DISCUSSION

As we all known, lipid accumulation in hepatocytes, liver fibrosis and intestinal barrier dysfunction showed up as NAFLD progresses.^18^, ^19^ Moreover, NAFLD has high prevalence in the world and correlates to an increased risk for development of diabetes.^20^ However, only limited therapeutic options are available. Genome‐wide association studies have discovered several genetic variants that involved in NAFLD, which helps us to identify individuals at high risk of suffering from NAFLD, but also to better understand its pathophysiology so as to develop more effective treatments; nonetheless, more prospective studies need to be done.^21^ In this study, we identified the functions of GRHL2 in NAFLD and further found that SIRT1 down‐regulation induced by GRHL2‐dependent miR‐200 or MAPK signalling pathway activated by GRHL2 exacerbated liver fibrosis and intestinal mucosal barrier dysfunction in NAFLD.

Initially, our data revealed that GRHL2 was up‐regulated in NAFLD mice. Various researches have widely identified the role of GRHL2 in liver diseases. For example, GRHL2 potently exacerbated liver injury and fibrosis by inhibiting miR‐122 expression in ethanol‐induced liver disease.^22^ The same mechanism was found to restrict the hepatocytic differentiation potential of adult liver progenitor cells.^8^ Hence, GRHLX, a transcription factor, promotes the progression of liver disease especially NAFLD.

Subsequently, another critical finding in our study was that miR‐200 was up‐regulated in NAFLD and GRHL2 exerts contributory role on NAFLD by up‐regulating miR‐200 expression. miRs have been pivotal regulators of metabolism including cholesterol and lipid metabolism, insulin and glucose homeostasis and hepatic lipid homeostasis.^23^ Furthermore, a recent study employed RNA sequencing to obtain hepatic expression profiles of mRNAs and miRs in NAFLD and normal rats, in which miR‐200 family (miR‐200b‐3p, miR‐200b‐5p, miR‐200c‐3p) were identified as elevated miRs in NAFLD.^24^ Consistently, a prior study indicated that miR‐200 family genes were positively regulated by GRHL2 in ovarian cancer by binding to miR‐200’ promoter or indirectly involving in other signalling pathways through transcriptional regulation.^9^ Also, GRHL2 promotes miR‐200 expression by directly binding at the miR‐200 promoter region, which leads to oral cancer development.^14^ Importantly, our study also portrayed that miR‐200 inhibition alleviated liver fibrosis and intestinal mucosal barrier dysfunction in NAFLD mice. Similarly, the overexpression of miR‐200 family was seen to exert influence on liver fibrosis and miR‐200 elevation induced the progression of liver fibrosis.^25^

Also, according to dual‐luciferase reporter gene assay, we elaborated that SIRT1 was targeted by miR‐200. Supportively, previous studies confirmed the targeting relationship between SIRT1 and miR‐200a in breast cancer.^13^ Interestingly, the results observed in the present study also indicated that SIRT1 elevation ameliorated liver fibrosis and intestinal mucosal barrier dysfunction in NAFLD mice. Likewise, the elevation of SIRT1 expression was found to be dramatically inhibited the inflammatory fibrosis.^26^ It is reported that SIRT1 was targeted by miR‐181b to regulate steatosis and down‐regulated in NAFLD.^27^ Another study further explored that ablated SIRT1 activity resulted in more severe NAFLD by affecting liver‐mesenteric adipose tissue fatty acid mobilization.^28^ In addition, a prior study also noted that SIRT1 exacerbates liver fibrosis in mice.^29^ Therefore, SIRT1, negatively regulated by miR‐200, played a vital role in promoting NAFLD progression.

Last but not least, the present study validated that GRHL2 activated MAPK signalling pathway and exacerbated liver fibrosis and intestinal mucosal barrier dysfunction in NAFLD mice. It has been documented that GRHL2 is contributed to conferring collecting duct epithelial barrier dysfunction, as evidence by diminished expression of tight junction–related barrier components.^30^, ^31^, ^32^ Accordingly, we proposed that GRHL2 may be involved in forming intestinal mucosal barrier because the intestinal mucosa is composed of epithelial cells. Moreover, consistent with our study, MAPK was also found to be activated by GRHL2 in oral cancer.^14^ And, p38 MAPK blocking was found in prior study to permit DNA replication during regeneration of liver and maintain the hepatocyte cell cycle arrest in adult liver.^14^, ^33^ Additionally, the inhibition of MAPK signalling pathway was involved in improved liver fibrosis ^34^ and the inhibition of phosphorylated MAPKs was highly hepatoprotective and anti‐inflammatory.^35^ Taken together, it can be inferred that inhibition of MAPK signalling pathway played a suppressive role in NAFLD development.

## 5 CONCLUSION

**FIGURE 6:**
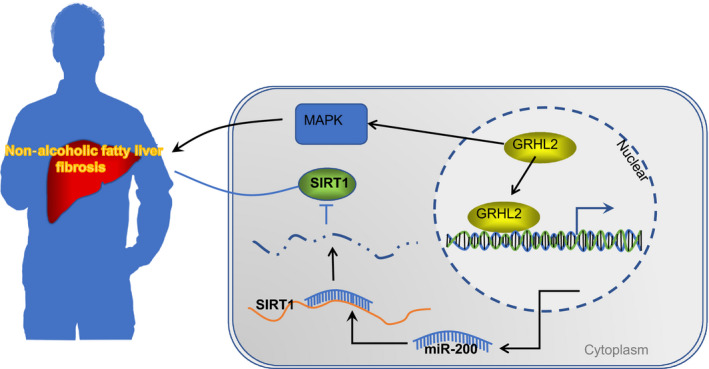
Mechanism of GRHL2‐dependent miR‐200 in NAFLD. GRHL2 exacerbates liver fibrosis and intestinal mucosal barrier dysfunction by down‐regulating SIRT1 through activating miR‐200. Meanwhile, GRHL2 also ameliorates liver fibrosis and intestinal mucosal barrier dysfunction by activating MAPK signalling pathway in NAFLD

In conclusion, the present study provides new insights into the mechanism underlying the non‐alcoholic fatty liver fibrosis and intestinal mucosal barrier dysfunction (Figure 6). Particularly, transcription factor GRHL2, which was up‐regulated in NAFLD patients and mouse models, could up‐regulate miR‐200 by binding to miR‐200 promoter, thereby inhibiting the expression of SIRT1. Meanwhile, GRHL2 knockdown ameliorated liver fibrosis and intestinal mucosal barrier dysfunction by inactivating MAPK signalling pathway in NAFLD mice. These two mechanisms made GRHL2 a therapeutic candidate for NAFLD. However, further studies need to be performed in the future due to the lack of reports about the relationship between GRHL2 and intestinal mucosal barrier dysfunction.

## CONFLICT OF INTEREST

The authors declare no competing interest.

## AUTHOR CONTRIBUTIONS

Ying Wang, Zishu Zeng wrote the paper and conceived and designed the experiments; Lin Guan, Ran Ao analysed the data; Zishu Zeng and Lin Guan collected and provided the sample for this study. All authors have read and approved the final submitted manuscript.

## Data Availability

The data that support the findings of this study are available from the corresponding author upon reasonable request.
