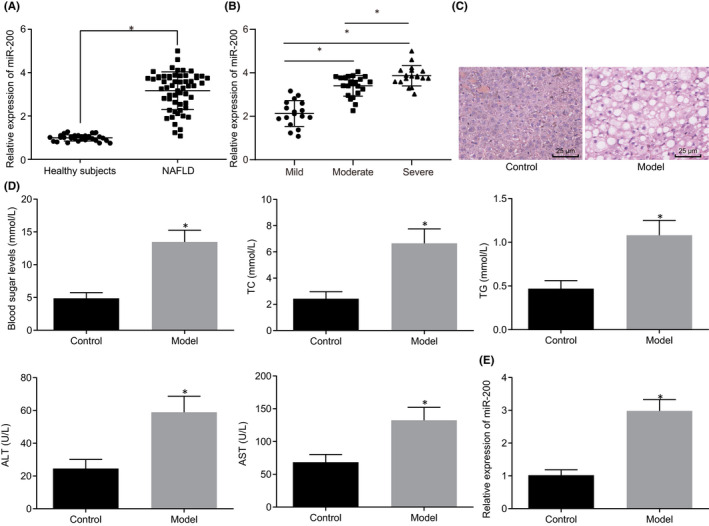# Corrigendum

**DOI:** 10.1111/jcmm.17393

**Published:** 2022-06-06

In Ying Wang et al,^1^ The ordinate of the third histogram in Figure 1D was incorrectly written. It was incorrectly written as TC, and the correct one should be TG. The correct Figure 1 are shown below. The authors confirm that all results and conclusions of this article remain unchanged.

**FIGURE 1:**